# A pilot study on the novel use of an ultrasonic toothbrush in dogs: limited effect on dental calculus following a single treatment

**DOI:** 10.1186/s13028-025-00843-4

**Published:** 2026-03-23

**Authors:** Karolina Brunius Enlund, Gunilla Andersson, Lina Dolfei, Molly Lavesson, Jenny Palm, Lena Olsén

**Affiliations:** Department of Clinical Sciences, Faculty of Veterinary Medicine and Animal Science, Swedish University of Agricultural Sciences, Box 7054, Uppsala, 750 07 Sweden

**Keywords:** Canine, Dental health, Tartar

## Abstract

**Background:**

Dental calculus is common in dogs and contributes to periodontal disease by promoting plaque accumulation. While daily tooth brushing is the gold standard for prevention, the gold standard for calculus removal is professional dental cleaning under anesthesia at a veterinary clinic. Ultrasonic toothbrushes are marketed as alternatives for single-session calculus removal while the dog is awake; however, there is currently no scientific evidence supporting their effectiveness for this purpose. The aim of the study was to evaluate the effectiveness of a single ultrasonic toothbrush session for canine dental calculus removal. The study consisted of two parts. In part one, beagle dogs with confirmed dental calculus underwent a single session of ultrasonic toothbrush application on the maxillary canine and maxillary fourth premolar, with six minutes per tooth. Stress levels were assessed using the FAS scale (Fear, Anxiety, and Stress). In part two, teeth with dental calculus from specimens were treated with the ultrasonic toothbrush for up to 40 min per tooth. A blinded assessor evaluated dental calculus index pre- and post-treatment.

**Results:**

No visible reduction in calculus was observed in the dogs. Stress during brushing was low (< 2 out of 5 on FAS-scale) and significantly lower (*P* < 0.05) for canine teeth compared to the fourth premolar. Among the specimens, 7 out of 10 teeth lost small pieces of calculus during mechanical dislodgement with a fingernail after 6–28 min of ultrasonic toothbrush treatment; however, only two teeth showed sufficient reduction to lower the calculus index.

**Conclusion:**

Ultrasonic toothbrushes show no significant impact on dental calculus removal as claimed by service providers. While extended use on a single area (> 10 min) may slightly weaken calculus, its clinical relevance is minimal. These brushes are effective for daily prevention of calculus formation but not for its removal once formed. Professional dental cleaning at a veterinary clinic is recommended to address calculus and ensure other dental issues are not overlooked.

## Background

Dental disease is very common in dogs, with periodontal disease affecting over 80% of dogs over 3 years of age [1–3]. Dental calculus, also known as tartar, is very common in dogs [4]. The development of dental calculus begins when plaque, a bacterial biofilm rich in oral bacteria, becomes mineralized with calcium phosphate salts. This mineralized biofilm forms both supragingivally and subgingivally and is always covered with a layer of viable microorganisms [5]. While calculus itself is not pathogenic, it facilitates increased plaque formation, which can initiate the inflammatory process of periodontal disease [6]. However, there is generally a low correlation between calculus and periodontitis [7].

Once formed, calculus becomes strongly bound to the tooth surface, requiring professional periodontal therapy under anesthesia, usually using an ultrasonic scaler and curette [4]. During this procedure, a veterinary dental examination is essential to detect and treat signs of dental disease. It is important to note that a single removal of visible calculus, regardless of the method, without subsequent daily tooth brushing, is primarily a cosmetic measure without lasting effects on inflammation or periodontal disease [3, 4].

The gold standard for dental care, both in preventing calculus and periodontal disease, is daily tooth brushing, in dogs as well as in humans [3]. Daily brushing removes the soft bacterial biofilm (plaque), preventing calculus formation.

An alternative to the traditional toothbrush is the ultrasonic toothbrush, which uses ultrasound to remove plaque and kill plaque-forming bacteria [8, 9]. Ultrasonic toothbrushes have been commercially available in human dentistry since 1992, when a patent for the technology was approved [8]. These toothbrushes generate high-frequency sound waves, ranging from 20 kHz up to 10 MHz depending on the manufacturer, and have the appearance of electric toothbrushes. Notably, they differ from the ultrasonic scalers used by veterinarians or dentists, which transmit vibrations via a metallic tip. Ultrasonic toothbrushes instead operate using the cavitation effect, in which sound waves cause the growth, oscillation, and collapse of gas bubbles in a liquid, removing deposits like biofilm and bacteria without applying pressure or moving the brush. The brush is held still and does not emit audible sound. The propagation of ultrasonic waves depends entirely on the medium, often consisting of water, saliva, and toothpaste [8].

With daily use in humans, ultrasonic toothbrushes are comparable to other powered toothbrushes in soft plaque removal, though they show an advantage over manual toothbrushes [8, 10]. However, manufacturers of ultrasonic toothbrushes for dogs claim that regular use can also reduce and remove calculus, and that similar results can be achieved through single-session treatments [11, 12]. Calculus removal treatments are also offered by dog groomers, dog salons, and dog daycares, where one or more teeth are treated with an ultrasonic toothbrush for up to 45 minutes to remove calculus [13–18]. Currently, there is no scientific evidence supporting this practice.

The aim of the study was to evaluate the effectiveness of a single ultrasonic toothbrush session for canine dental calculus removal. This was achieved by:

1. Live animal study: To investigate whether a single session of ultrasonic tooth brushing, lasting 6 min on a single tooth, can eliminate visible dental calculus, and whether the dog shows signs of stress during the treatment.
2. Specimen trial: To investigate whether a single session of ultrasonic tooth brushing, lasting up to 40 min on a single tooth, can eliminate visible dental calculus, and, if so, to determine how long it takes for any visible reduction to occur.

## Methods

### Study design study 1

Twenty-nine beagle dogs, housed and used as teaching dogs at the Swedish University of Agricultural Sciences (SLU) in Uppsala, were assessed by author KBE for eligibility. Dogs without visible calculus, with dental fractures of the maxillary canine or maxillary fourth premolar, or a noticeable difference in the amount of calculus between the right and left sides were excluded. This resulted in seventeen beagle dogs being included in the study.

**Fig. 1:**
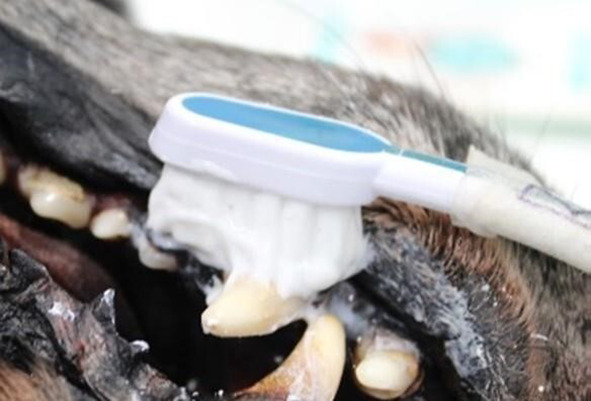
The use of an ultrasonic toothbrush with toothpaste in this study.

The toothbrush used was the Emmipet Ultrasonic toothbrush (emmi^®^-pet, Emmi-ultrasonic, Germany) with separate toothbrush heads for each dog, model A2 (emmi^®^-pet, Emmi-ultrasonic, Mörfelden-Walldorf, Germany), along with a special toothpaste (emmi^®^-pet, Emmi-ultrasonic, Mörfelden-Walldorf, Germany) according to the manufacturer’s recommendation (Fig. 1) [11].

Assessment of dental calculus was performed pre-and post-treatment by author KBE (Table 1) [4].

**Table 1:** Calculus index (CI) used in both studies (4)

0	No calculus
1	Supragingival or calculus that extends only slightly below the free gingival margin
2	Moderate amount of supra- and/or subgingival calculus or only subgingival calculus
3	Abundant supragingival and/or subgingival calculus

The calculus on the maxillary canine and maxillary fourth premolar on either the left or right side was treated with the ultrasonic toothbrush and special toothpaste for six minutes per tooth. The brush was applied without pressure or movement to the calculus surface, following the user manual. At one-minute intervals, the calculus was manually scraped three times using a fingernail. Any dislodged material was removed, and toothpaste was re-applied. The dogs were randomized as to which side of the maxilla was to be treated: nine dogs were treated on the left side, and eight dogs were treated on the right side. In alternating dogs, the maxillary canine was treated first, while in the others, the maxillary fourth premolar was treated first. The buccal sides of the maxillary canine and maxillary fourth premolar were assessed using photographs taken before and after treatment. The untreated side served as a control to blind the assessor.

The dogs’ stress levels were assessed by authors GA and JP during tooth brushing, using the five-point Fear Anxiety Stress (FAS) scale [19], before treatment began and after 1, 3, and 6 min of treatment. The scale primarily describes behavioral and physiological changes exhibited by the dogs during fear, anxiety and stress, categorizing them into three levels: low (0–1), moderate (2–3), and high (4–5) [19]. Differences in mean FAS between the treatment of the maxillary canine and the maxillary fourth premolar, as well as differences in mean FAS between time-points in treatment, were investigated using a paired two-sided t-test, with statistical significance set at *P* < 0.05.

The dogs and facilities used in the study were approved by the Ethics Committee for Animal Experimentation, Uppsala, Sweden (Approval No: Dnr 5.2.18–7454/15, User permit: Dnr 5.2.18–2636/15, Teaching permit: Dnr 5.8.18–15533/2018). The study did not require additional approval as no invasive procedures were performed.

## Study design study 2

Euthanized dogs (*n* = 5), donated by their owners, were used in the study, with written consent for research and education obtained. The dogs were of different breeds and ages, and the inclusion criteria were the presence of calculus (CI1-3; Table 1) [4] on the maxillary canines and maxillary fourth premolars. Specimens had been frozen and were thawed for the study. On each specimen, the maxillary canine and maxillary fourth premolar on one side of the jaw (teeth; *n* = 10) were treated with the same ultrasonic toothbrush and toothpaste as in study 1 (emmi^®^-pet), by holding the toothbrush head in the same position on the calculus (Fig. 1). Toothpaste was reapplied if the toothbrush began to appear dry. Treatment was discontinued once all calculus had been removed or the maximum treatment time of 40 min had been reached. At two-minute intervals, the calculus was manually scraped, by the same person, three times using a fingernail. Assessment of dental calculus was performed pre- and post-treatment by a blinded assessor (author KBE).

## Results

### Study 1

#### Calculus

**Fig. 2:**
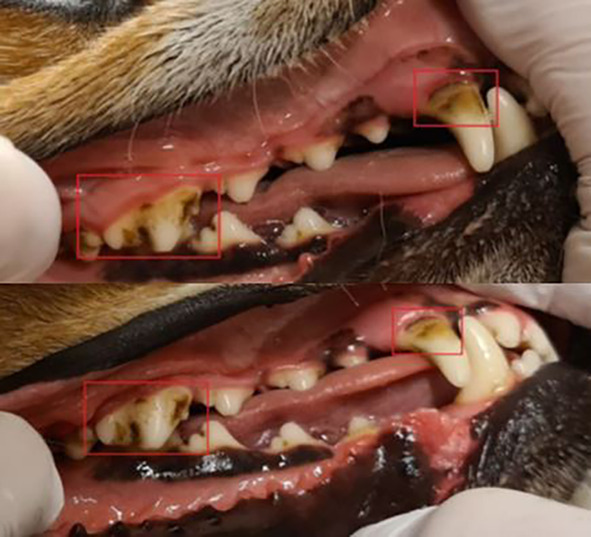
Dental calculus in adog before and after ultrasonic brushing. Dental calculus in a dog from study 1 before treatment (upper figure), and after 6 min of treatment with the ultrasonic toothbrush on the maxillary fourth premolar and 6 min on the maxillary canine tooth (lower figure, squares)

Before treatment, the mean calculus index (CI) was 2.2. Four dogs had a CI of 3, and 13 dogs had a CI of 2. The calculus index did not differ between the left and right sides within the same individual, as specified in the inclusion criteria. After treatment, no improvement was detected in any dog by the blinded assessor, and the mean calculus index remained the same at 2.2. For example, see Fig. 2.

#### FAS

**Fig. 3:**
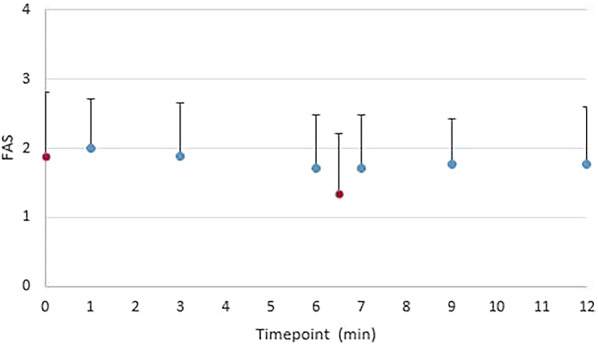
Time course of FAS (Fear, Anxiety, and Stress) in dogs during ultrasonic tooth brushing treatment. Mean values and SD for the assessed FAS levels in dogs (*n* = 17) during the treatment of the maxillary fourth premolar (6 min) and maxillary canine teeth (6 min) with an ultrasonic toothbrush for a total of 12 min. In every other dog (*n* = 8), the maxillary fourth premolar was treated first, while in the others (*n* = 9), the canine tooth was treated first. Red dots indicate time points when no treatment is taking place (before start and during the change of tooth, significant difference in FAS *P* < 0.05)

The dogs’ mean FAS value was 1.88 before treatment started, and 1.76 at the end of the treatment of the second tooth, after 12 min (Fig. 3). The FAS level was significantly lower only during the pause between treatments (time 6.5) compared to before treatment (time 0), i.e., lower during the pause between the treatment of the maxillary canine and maxillary fourth premolar (*P* < 0.05; Fig. 3).

**Fig. 4:**
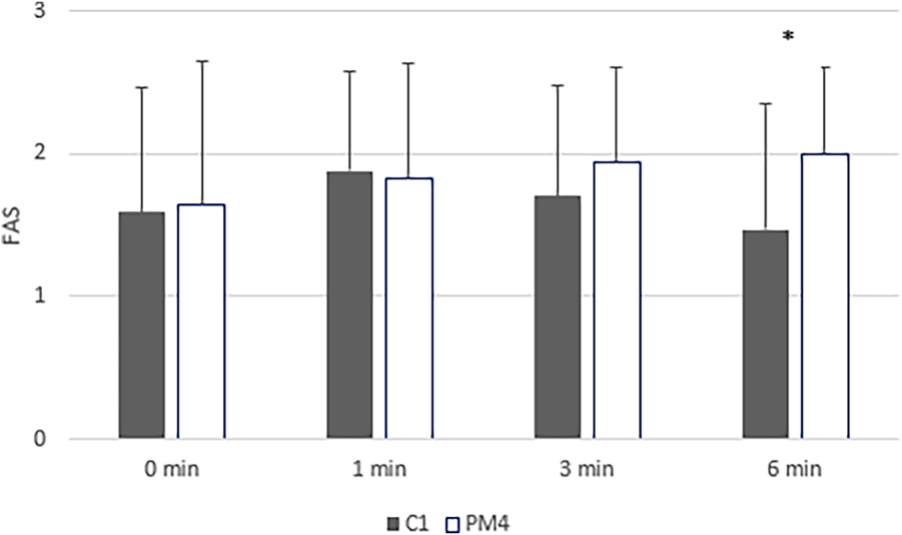
FAS (Fear, Anxiety, and Stress) levels by tooth in dogs during ultrasonic tooth brushing treatment. Mean FAS value for dogs (*n* = 17), assessed 1, 3, and 6 min into treatment of maxillary canine tooth and maxillary fourth premolar, respectively. In every other dog (*n* = 8), the maxillary fourth premolar was treated first, while in the others (*n* = 9), the canine tooth was treated first. * significant difference (*P* < 0.05)

The dogs’ FAS level was also significantly lower at the end of the canine tooth treatment (1.47) compared to the end of the fourth premolar treatment (2.00; *P* < 0.05; Fig. 4). The dogs’ FAS level also decreased significantly (*P* < 0.05) during the canine tooth treatment (from 1.88 to 1.47), while it increased slightly during the treatment of the fourth premolar (from 1.82 to 2.00), though this increase was not significant (Fig. 4). Some dogs (*n* = 7) were assessed to have a higher FAS level at the beginning, while others (*n* = 4) were assessed to have a higher FAS level towards the end of the treatment, regardless of tooth.

### Study 2

#### Calculus

**Fig. 5:**
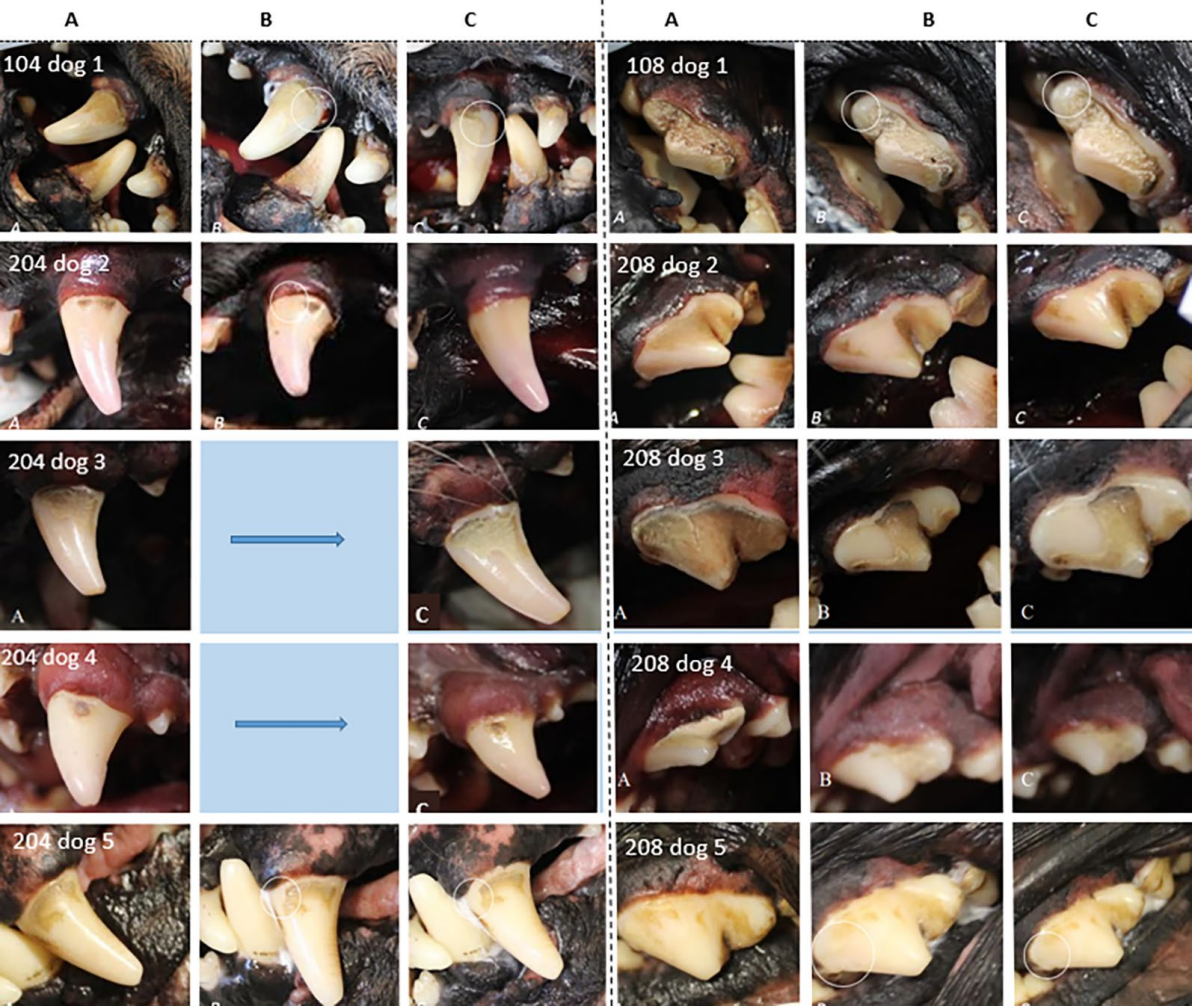
Results for specimen teeth, photographs. **A** Shows before treatment, **B** at the first visible difference (if any), and **C** results at maximum treatment time reached. Note 1: 208 on dog 3, the piece of calculus was removed by scraping before the ultrasonic toothbrush was first applied. Note 2: The post-treatment increase in space between gingival margin and calculus observed in the photographs was explained by the mechanical influence of the brush on the specimens’ gingival margin, as it is evident in the photographs that no calculus had been removed

In the specimens, before treatment the mean calculus index (CI) was 2.1. Seven teeth out of ten lost a small piece of calculus during scraping with fingernail after 6–28 min of treatment with ultrasonic toothbrush (Table 2), although only two of them lost enough to lower CI. No calculus detached by itself without manipulation. One tooth lost calculus during scraping even before treatment commenced, this tooth also lowered its calculus index. In two teeth, there was no difference at all after 40 min of treatment (Table 2; Fig. 5).

**Table 2:** Results for specimen teeth (*n* = 10)

Specimen	Tooth	Calculus index pre-treatment	Calculus index post-treatment	Time to first noticeble difference (min)	Total treatment time (max 40 min)
1	104	2	2	20	40
	108	3	3	18	40
2	204	1	0	10	10
	208	3	2	20	40
3	204	2	2	No diff	40
	208	3	2	0*	40
4	204	1	1	No diff	40
	208	2	2	28	40
5	204	2	2	6	40
	208	2	2	16	40

Presentation of calculus index, the first visual difference (small piece of calculus detached) inthe amount of calculus, and total treatment time.*A piece of calculus was removed by scrapingbefore the ultrasonic toothbrush was first applied. No further calculus was lost during treatment

## Discussion

The study showed that treatment with an ultrasonic toothbrush on a single occasion is insufficient to remove a clinically relevant amount of calculus. Potentially, the calculus may weaken slightly, as in some specimens (Fig. 5), small pieces of calculus could be removed with a fingernail after up to 28 min of treatment with the ultrasonic toothbrush in a single spot. In only one specimen (both teeth), sufficient calculus detached to reduce the calculus index. However, no calculus detached without scraping. The potential weakening should be regarded as having little to no clinical significance, as the pieces removed were small, and the extremely long treatment time on a single tooth in a live animal is neither practical nor ethical, for humans or dogs. As dog groomers and similar practitioners are marketing this practice to dog owners as a safe and effective way of removing calculus, this may deter owners from seeking necessary veterinary care for their dogs.

In humans, the rate at which dental calculus forms varies significantly from person to person but tends to be consistent for an individual [5]. This process is influenced by several factors, such as diet (particularly the intake of alkaline foods and sugars), genetic differences in saliva composition, and other variables like age, gender, health conditions, and the amount of bacteria present. However, there is still limited understanding of how quickly calculus builds up over a lifetime or whether it can be dissolved. While the composition of human dental calculus has been extensively researched [5, 20, 21], the composition and formation of dental calculus in dogs remain scarcely studied [22, 23].

There are both similarities and differences between dental calculus in dogs and humans. The dental calculus of dogs consists primarily of calcium carbonate with a small amount of apatite, whereas other calcium phosphates common in humans are not present in dog calculus [22]. This difference is also reflected in the saliva, where calcium carbonate predominates in dogs, and apatite in humans, respectively [22]. One study showed that salivary pH, buffering capacity, and concentrations of Ca, K, and Na are higher in dogs than in human saliva [24]. In contrast, the concentration of P was lower compared to the average levels found in human saliva [24]. Furthermore, differences in the salivary proteomic profile have been observed in dogs with and without dental calculus, highlighting the role of salivary proteins associated with dental calculus and the complexity behind dental issues [25].

Additionally, dogs generally have a higher pH, 8–8.5 [24, 26], in their saliva than humans, pH 6–7 [27], which contributes to the formation of dental calculus. Calcium salts are more likely to precipitate in a more alkaline environment. This difference in pH may also help explain why dental caries is much less common in dogs than in humans [24].

In one study, the saliva of dogs from different breeds was examined [24]. Buffer capacity and pH values were comparable for tested breeds, except for Labrador retrievers. Compared to dachshunds and Jack Russell terriers, Labrador retrievers had higher concentrations of P, Ca, Na, and K. Although the amount of calculus was not reported in the study, these differences in mineral composition and physiological conditions may partly explain why different breeds exhibit varying degrees of dental calculus [24]. Other contributing factors to rapid calculus formation could include the dog’s level of salivary phosphorus [28], occlusion, including crowding of teeth, and amount of saliva. In our study, beagle dogs were used in the first part and different breeds in the second. It is unknown how their calculus composition influenced the results.

In humans, a distinction is made between supragingival and subgingival calculus. Subgingival calculus originates from the gingival crevicular fluid, forms below the gumline, and is generally harder, more tenacious, and darker than supragingival calculus [20]. Supragingival calculus forms from minerals and proteins in saliva, and is generally softer, lighter in color, and easier to remove than subgingival calculus [20]. Few or no scientific studies have reported on differences in calculus composition in dogs, although such differences are considered highly likely, and also seen in our study. In our second study, specimens from different breeds were examined, and although the sample size was limited, macroscopically different types of calculus were observed (Fig. 5). This is also commonly seen in clinical practice, where the structure, color, or difficulty in removing dental calculus varies widely between individuals. Although based on a single individual, the fact that specimen no. 2 showed a reduced calculus index on both treated teeth may indicate individual differences in calculus formation and attachment to enamel.

The gold standard for dental calculus removal in dogs is a professional dental cleaning at a veterinary clinic, with the dog under anesthesia, using an ultrasonic scaler and hand instruments [3]. This procedure includes a full dental examination, including dental radiography, to detect any underlying pathology. Removing visible calculus from an awake dog may give a false sense of security, causing dog owners to postpone or cancel a veterinary visit, potentially leaving dental pathology undetected and untreated. Using a dental hand scaler on an awake dog is also discouraged due to the risk of injury to both the dog (to the enamel and soft tissue) and the person performing the procedure [3]. When interviewing providers of single-session ultrasonic tooth brushing for calculus removal, it became evident that many also use a dental hand scaler as a supplement—an often used method for calculus removal even without prior treatment. Others recommend giving the dog a bone to chew on after the procedure. It is unknown how this affects the calculus, but if long-term treatment with the ultrasonic toothbrush on a single tooth weakens the calculus, it could contribute to some calculus detaching after treatment.

To prevent dental disease in the dog, the focus should be on daily dental home care [3], where an ultrasonic toothbrush may be one of many options. Other options for so-called active daily dental care include manual toothbrushes, electric toothbrushes, or using textiles made of microfiber or nylon, for example [9]. As a complement, owners may choose to use passive dental home care products, some of which have shown some effect in clinical trials [29].

Ultrasonic toothbrushes have been shown to have largely the same efficacy on dental plaque as electric toothbrushes with daily use [8, 9, 30]. However, one study showed significantly better results with daily use of the ultrasonic toothbrush than with a manual toothbrush [10].

The manufacturer of one ultrasonic toothbrush claims that sound waves penetrate up to 12 mm into the gums and can reach gum pockets and cavities in the teeth [11]. However, this claim is not scientifically proven, to the authors’ knowledge. If accurate, it would represent a revolution in periodontitis treatment, as even deep periodontal pockets could be cleaned daily. As mentioned, there is no scientific evidence supporting this claim in either human or veterinary dentistry, and it should therefore be regarded as false advertising.

An in vitro study on human teeth showed that daily ultrasonic tooth brushing for two minutes over a period of two months did not cause any damage to the enamel [31]. There are currently no published studies investigating the potential harmful effects of using an ultrasonic toothbrush on dogs’ teeth, or the effects of longer treatment durations. Only one study has evaluated the use of an ultrasonic toothbrush in dogs for daily plaque removal [9]. The longer treatment times (up to 45 min) offered by dog groomers at a single session differ from the daily use of an ultrasonic toothbrush, and there is no scientific description or recommendation for this practice.

### FAS

The majority of the beagle dogs participating in the study had previously been involved in studies investigating dental home care procedures [9, 32] and thus had some prior experience, although no dental home care had been performed in the past year. In the previous study, the FAS score decreased significantly after five weeks of daily dental home care, demonstrating the dogs’ adaptation to the routine [9]. Although the FAS at the start of treatment in the present study was approximately the same as in the previous study [9], this may have influenced the relatively low stress levels observed during the longer treatment period in this study. Another factor may be that the brush is relatively easy for the dogs to accept, as it remains still with no motion or vibration and the brush does not seem to cause discomfort.

Interestingly, the treatment of the maxillary canine resulted in significantly lower FAS scores at the end of the treatment compared to the maxillary fourth premolar, regardless of which tooth was treated first. This aligns with clinical observations that dogs tend to react more strongly when manipulations occur further back in the oral cavity compared to the rostral part.

### Methodological considerations

The study is limited by the number of dogs and specimens.

Mechanical dislodgement with a fingernail was used as it reflects a technique occasionally used in clinical practice and provided a simple means to standardize the applied force across dogs and specimens.

The specimens were frozen after euthanasia and then thawed, and it cannot be ruled out that this process may have affected the dental calculus.

The assessment of calculus, even when using a scale, is subjective. One strength of the study, however, is the blinding of the assessor (author KBE), which minimizes assessment bias.

### Future studies

Few or no scientific publications have previously investigated if ultrasonic toothbrushes may weaken or remove dental calculus. However, a few recent publications within human dentistry have been published regarding radiofrequency (RF, 3 kHz till 300 GHz) toothbrush and calculus removal, although showing conflicting results [33–35]. Microcurrent-emitting toothbrushes, using so called bioelectric effect, have also been suggested as beneficial for plaque-removal [36], however, no or few studies regarding calculus have been published.

To further investigate if the calculus was weakened in some way by the treatment with ultrasonic toothbrush, histological studies of calculus before and after treatment would be of value.

## Conclusion

When it comes to single-use treatments with an ultrasonic toothbrush, we see no evidence of the spectacular effect on dental calculus that is promised by the service providers. We cannot completely disregard the possibility that calculus may weaken slightly with prolonged application on a specific area (> 10 min), but the clinical significance of this is considered low. The ultrasonic toothbrush is effective with daily use for preventing plaque accumulation and calculus formation, but once calculus has formed, it is not an effective method for removing it. In such cases, professional dental cleaning at a veterinary clinic is recommended. This is also important to ensure that dental pathologies do not go undetected.

## Data Availability

Data available from corresponding author on request.
